# Acidification of colostrum affects the fecal microbiota of preweaning dairy calves

**DOI:** 10.3168/jdsc.2022-0296

**Published:** 2023-02-09

**Authors:** Meagan Hennessy, Michaela Kristula, Sarah Cady, Billy Smith, Nagaraju Indugu, Bonnie Vecchiarelli, Dipti Pitta

**Affiliations:** Department of Clinical Studies, School of Veterinary Medicine, University of Pennsylvania, New Bolton Center, Kennett Square 19348

## Abstract

Calf diarrhea is a leading cause of death in preweaning calves and it causes major economic losses to producers. Acidified milk has been shown to have beneficial effects on health and growth parameters in calves but there is little research into its effects on the microbiota, and few studies on the use of acidified colostrum. The purpose of this study was to compare how feeding acidified colostrum to calves at birth affects fecal microbiota from birth through 8 wk of age compared with calves fed nonacidified colostrum. In this study, 5 calves received acidified colostrum (treated group) and 5 calves received nonacidified colostrum (control group) at birth and at 12 h of age. All calves were subsequently fed acidified whole milk until weaning at 8 wk of age and had access to starter grain starting at d 3 and throughout the study. Fecal samples were collected at 24 h, 48 h, and at 1, 2, 3, 4, 5, 6, 7, and 8 wk of age. Samples were extracted for genomic DNA, PCR-amplified for the V1-V2 region of the 16S rRNA bacteria gene, sequenced, and analyzed using QIIME2. Bacterial richness (estimated by number of observed species) and bacterial diversity (estimated by Shannon diversity index) differed between time points but not between treatment groups, and both increased over time. Weighted and unweighted UniFrac analysis showed differences between bacterial communities across time points and treatments. Across all time points (lmer test), 6 bacterial genera were different between treatments: *Faecalibacterium* and unclassified *Clostridiaceae* were more abundant, whereas *Atopobium*, *Collinsella*, *CF231*, and unclassified *Veillonellaceae* were less abundant in treated versus control calves. *Faecalibacterium* is a butyrate-producing bacterium that has been linked to decreased prevalence of diarrhea in calves. Our results indicate that there is considerable flux in the calf microbiome through the neonatal period and weaning transition but that feeding acidified colostrum followed by acidified whole milk allowed early colonization of *Faecalibacterium*. Further studies are needed to verify the positive benefits of promoting *Faecalibacterium* on improving the health of preweaning calves.

## Highlights

• Acidifying milk and colostrum may improve dairy calf health.
• Little research has been conducted on how this affects gastrointestinal microbiota.
• Acidifying colostrum increased abundance of *Faecalibacterium* at 1 wk of age.
• Acidified colostrum affects calf microbiota.

## Graphical Abstract

**Figure:**
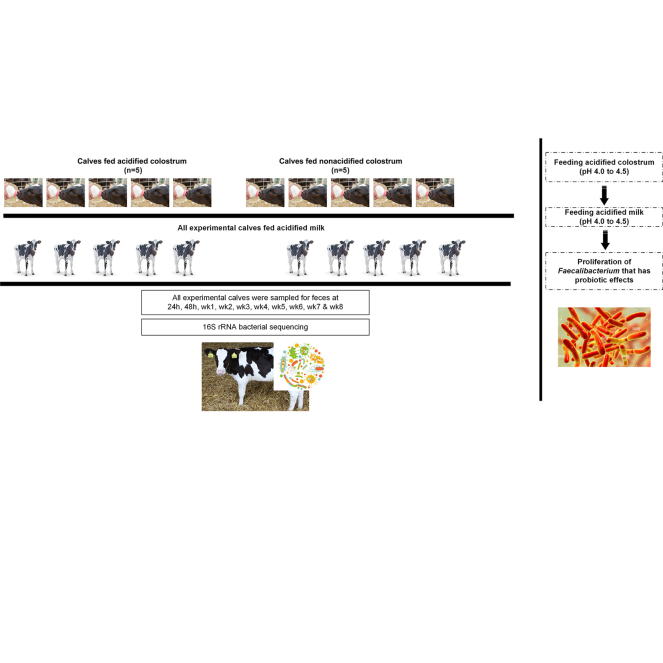
**Summary:** Diarrhea is a leading cause of death in preweaning calves. Acidification of colostrum is an effective tool in colostrum management. The goal of this study is to investigate the effect of feeding acidified colostrum to calves at birth on fecal microbiota during the preweaning period compared with calves fed nonacidified colostrum. Findings of this study indicated a considerable flux in the calf microbiome through the neonatal period and weaning transition. Feeding calves with acidified colostrum induced changes only in a few fecal bacteria but allowed for early colonization of *Faecalibacterium* compared with control calves. *Faecalibacterium* is a butyrate-producing bacterium that is associated with decreased diarrhea and improved growth in calves.

Feeding calves to their nutritional requirements is critical to their growth and development during the preweaning period and it has a significant effect on production efficiency later in life. Feeding an adequate amount of good-quality colostrum early is essential to limit exposure to bacterial pathogens and ensure the health and survival of calves (Lorenz et al., 2011; Godden et al., 2019). However, colostrum can also expose calves to pathogenic microorganisms that can increase their risk of disease and decrease the efficacy of antibody absorption (Gelsinger et al., 2015; Mellado et al., 2017). Preservation of colostrum and milk is necessary to increase shelf life while minimizing bacterial loads and maximizing the availability of immunoglobulins needed for calf growth and development (Godden et al., 2003). The most commonly used preservation method is pasteurization, which has demonstrated effects on improving weight gains and reducing mortality and morbidity (Jamaluddin et al., 1996; Armengol and Fraile, 2016, 2020). Acidification of milk, typically by the addition of formic acid, is another preservation method that decreases bacterial loads by lowering the pH to 4.0 to 4.5 (Collings et al., 2011; Todd et al., 2018). This method is a viable option to increase the shelf life of milk without altering its nutritional benefits (Anderson, 2008). Some studies have reported that feeding acidified milk or acidified milk replacer to calves led to improved weight gain and better fecal scores, which are good indicators of calf growth and development (Li et al., 2019; Chen et al., 2020). However, further studies are needed to demonstrate improved health and performance when calves are fed acidified milk or acidified colostrum. In the United States, the use of either citric acid or potassium sorbate, but not formic acid, is allowed by the Food and Drug Administration (FDA) as a preservative for milk or colostrum for calves. Recently, Smith et al. (2022) used formic acid to acidify colostrum (<0.45%) and reported no detrimental effects of feeding acidified colostrum and milk in study calves. Several farms use formic acid-treated milk to feed their calves; however, the associated benefits on calf performance remain to be explored.

The microbiome (the population of microorganisms) begins to colonize the gastrointestinal tract soon after birth and it continues to evolve and adapt to changes in diet and environmental conditions from birth through weaning (Uyeno et al., 2010; Malmuthuge et al., 2014). A healthy core microbiome is essential to calf growth and development, and several reports have described the gut microbial community composition in dairy calves at various stages during the neonatal period (Oikonomou et al., 2013; Slanzon et al., 2022). Calf diarrhea is a common problem observed in the early stages of life, the cause of which is often difficult to determine (Cho and Yoon, 2014; Wei et al., 2021). Previously, we reported that calf diarrhea is associated with a loss of commensal bacteria, which are replaced with opportunists and pathogens compared with calves that did not experience diarrhea (Hennessy et al., 2021). The purpose of the current study was to investigate the effect of feeding acidified colostrum to calves at birth on fecal microbial profiles from birth through weaning. We hypothesized that feeding calves with acidified colostrum would induce early colonization of beneficial bacterial populations that may support health and growth during the neonatal period.

All animal studies were approved by the University of Pennsylvania Institutional Animal Care and Use Committee (IACUC protocol #807091). This study was conducted at a 500-head dairy farm (Nottingham, PA) between January and March 2020. Ten female Holstein dairy calves were enrolled in the study at birth as pairs born <12 h apart; 1 calf from each pair was assigned to the acidified colostrum group (**AC**; n = 5), and the other calf from each pair was assigned to the nonacidified colostrum group (control; n = 5). All calves received a total of 5.7 L of acidified or nonacidified colostrum, with 3.8 L given within 1 h of birth and 1.9 L given at their next feeding 12 h later. All calves were bottle fed and consumed the entire volume of each feeding. Colostrum was pooled from 2 animals for each of the study pairs. Immediately after milking colostrum from 2 fresh cows into clean stainless-steel milking buckets, the colostrum was measured for quality using a Hanna wine refractometer. Only colostrum that measured >24% Brix was selected for pooling. The colostrum was blended using a stainless-steel whisk, and 11.34 L was transferred to clean 2-quart (2 quarts equals 1.89 L) nursing bottles. The bottles were immediately chilled in a chest freezer for 12 h before transfer to a 2.2°C refrigerator. This ensured that there was adequate colostrum of appropriate quality for each pair of study calves before birth. If not used within 3 d of collection, the colostrum was discarded. The colostrum was acidified immediately before feeding using 9% formic acid to a target pH of 4.0 to 4.5. The initial 4 quarts fed after birth was acidified while at −16.5°C, and then warmed in a water bath to 40.6°C and fed by nipple bottle. This process was repeated 12 h later with the 2-quart second feeding. The pH of every aliquot of colostrum was measured using Hydrion pH indicator strips.

Birth weights of the calves ranged from 38 to 41 kg. Fecal samples were obtained from all calves at the following time points: 24 and 48 h, and 1, 2, 3, 4, 5, 6, 7, and 8 wk old, for a total of 10 time points for each of the 10 calves (100 samples). Based on balanced one-way ANOVA power calculation with treatments (k) = 2, sample size (n) = 5, and 10 sampling times, a total of 100 fecal samples were obtained during the experimental period, which provided power = 0.99, indicating adequate sample size for microbial diversity analysis. Samples were obtained via rectal stimulation, placed into plastic deli containers, and transported on ice to the laboratory, where they were stored at −20°C until DNA extraction.

During the course of the study, calves were housed outdoors in groups of 6, with access to a roofed 3-sided shelter and a fenced yard area. Calves were not separated by treatment group. For the duration of the study, all calves were fed acidified whole milk (acidified with 9% formic acid to a target pH of 4.0–4.5). The calves were weaned at 8 wk of age. Calves began to be offered free-choice starter grain at 3 d of age, which was available to them for the remainder of the study. No medications were administered to the calves at any point during the study. Free-choice water was available to the calves at all times.

Genomic DNA was extracted from 250 mg of each fecal sample using the repeated bead-beating and column method, followed by extraction with a commercial kit (QIAmp Fast DNA Stool Mini Kit; Qiagen Sciences) as described in Yu and Morrison (2004). For samples obtained at 24 and 48 h, an additional lysis step was added to the protocol because many of these samples consisted of meconium, which has low biomass and thus low DNA yield (Stinson et al., 2018). Briefly, for these samples, 1 mL of 10% Tween 80 detergent (EMD Millipore Corp.) was added to 250 mg of fecal material, bead-beaten using a Mini Bead Beater-16 (BioSpec Products Inc.) for 2 cycles of 3 min bead-beating/2 min icing, and centrifuged at 24,400 × *g* for 20 min at 4°C. Supernatant was discarded and the pellet resuspended in 1 mL of molecular-biology grade water and centrifuged at 24,400 × *g* for 20 min at 4°C. Supernatant was again discarded and the pellet used for extraction using the same protocol as samples from the other time points.

The extracted DNA was PCR-amplified, sequenced, and analyzed for 16S rRNA bacterial diversity as described in Hennessy et al. (2020, 2021). The α diversity matrices were compared between the treatment groups and sampling time points using linear mixed effect model (lmer) and Wilcoxon rank sum test. A nonparametric permutational multivariate ANOVA test (**PERMANOVA**; Anderson, 2001), implemented in the vegan package for R, was used to test the effects of day of sampling and treatment on overall community composition, as measured by weighted and unweighted UniFrac distance. The raw read counts from the 16S rRNA amplicon sequence variant (**ASV**) abundance table were collapsed at taxonomic rank and compositionally normalized (relative abundance) such that each sample summed to 1. The lmer test was used to test the differences in bacterial taxa between treatments (control and AC) groups, sampling points (24 and 48 h, 1, 2, 3, 4, 5, 6, 7, and 8 wk) and their interaction. The fecal microbiota sequences were deposited in the National Center for Biotechnology Information data set under BioProject accession number PRJNA843323.

For bacterial communities, approximately 3 million raw partial 16S rRNA sequences were obtained from 100 samples, with an average of 37,046 reads per sample (range: 12,402 to 53,566 reads). Quality filtering and denoising of these raw reads produced a total of 5,995 ASV. Fewer than 100 reads per sample were observed in the negative control samples and they were eliminated from further analysis.

To analyze α diversity or within-sample variation, individual results from the 10 calves were grouped by treatment for analysis of the number of bacterial species (ASV, estimated by species richness) and the distribution of bacterial species within each community (estimated by Shannon diversity index). No difference was observed between treatments (species richness: *P* = 0.914; Shannon diversity: *P* = 0.386; lmer), but both indices increased with age in both treatment groups (Figure 1). In β diversity, there was a significant difference observed between treatments and time points in both unweighted (treatment: *P* = 0.001; time point: *P* = 0.001; PERMANOVA) and weighted UniFrac (treatment: *P* = 0.001; time point: *P* = 0.001; PERMANOVA) matrices; however, the treatment difference were very small (weighted: R^2^ = 0.007; unweighted: R^2^ = 0.009) compared with the differences observed between sampling time points (weighted: R^2^ = 0.518; unweighted: R^2^ = 0.444; Figure 2).

**Figure 1:**
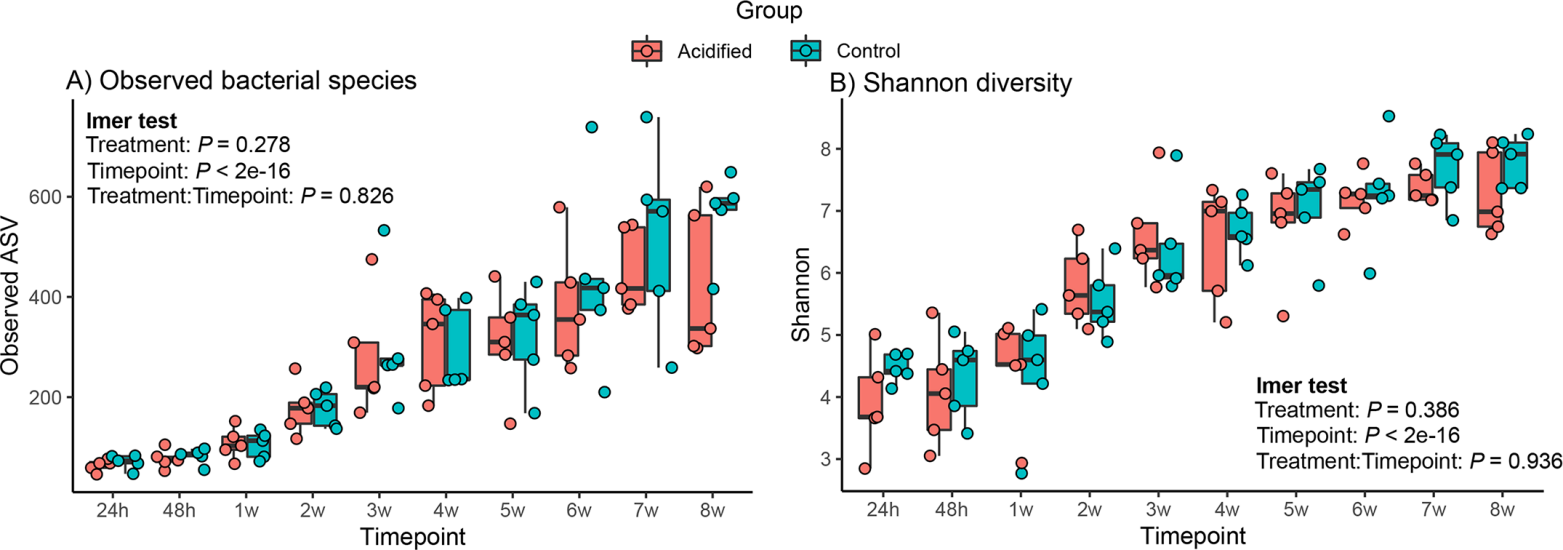
Alpha diversity or within-sample variation. Individual results from the 10 calves were grouped by treatment for analysis of (A) the number of bacterial species (amplicon sequence variant, ASV; estimated by species richness), and (B) the distribution of bacterial species within each community (estimated by Shannon diversity index). Boxes represent the interquartile range (IQR) between the first and third quartiles (25th and 75th percentiles, respectively), and the horizontal line inside the box defines the median. Whiskers represent the lowest and highest values within 1.5 × IQR from the first and third quartiles, respectively. w = week.

**Figure 2:**
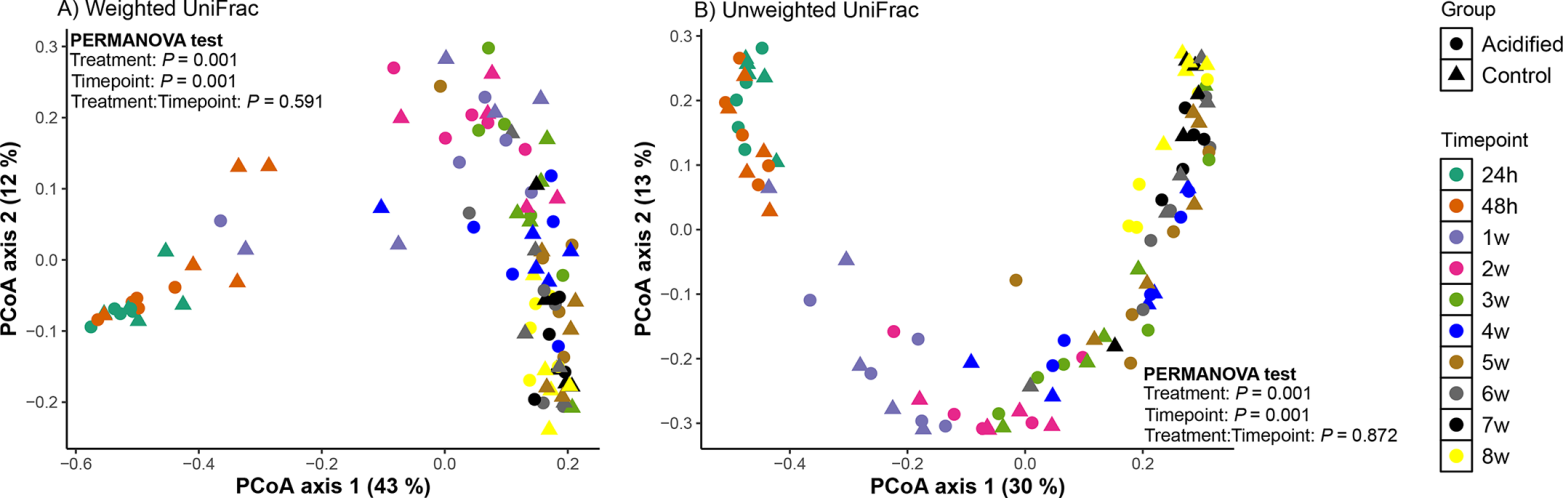
Beta diversity or between-bacterial community comparison by principal coordinate analysis (PCoA). Individual results from the 10 calves were grouped by treatment for analysis of (A) weighted and (B) unweighted UniFrac (permutational multivariate ANOVA, PERMANOVA) matrices. w = week.

At the genus level, the 24- and 48-h fecal samples were predominated by members of *Lactobacillales*, *Clostridiales*, and *Enterobacteriales*, which varied in relative abundance between the 2 groups. In the AC group at wk 1, the dominant players were *Enterococcus*, *Fusobacterium*, *Bacteroides*, *Faecalibacterium*, *Blautia*, *Dorea*, and *Collinsella*, whereas *Enterococcus* and *Faecalibacterium* were numerically less abundant in the control group. At wk 1, both *Collinsella* and *Clostridium* genera predominated in the control group, whereas their relative abundance was numerically lower in the AC group. From wk 2 through wk 5, *Blautia* and *Dorea* were dominant in the AC group and numerically less abundant in the control group. In contrast, unclassified *Ruminococcaceae* and *Streptococcus* were numerically more abundant in the control group. Interestingly, the relative abundance of *Faecalibacterium* was similar between the 2 groups during this period. From wk 6 to 8, the relative abundance of dominant genera including *Ruminococcus*, *Clostridiales*, *Lachnospiraceae*, and *Bacteroides* remained similar between the 2 groups.

Based on lmer analysis, most bacterial populations showed differences by sampling points (data not shown), and only 6 bacterial lineages at the genus level were significantly different between the AC and control groups across all sampling times (Figure 3). Of these, 4 genera, *Atopobium* (*P* = 0.003), *Collinsella* (*P* = 0.017), *CF231* (*P* = 0.007), and unclassified *Veillonellaceae* (*P* = 0.028), were more abundant in the control group in at least 3 sampling time points. Across all sampling points, the mean relative abundance of *Faecalibacterium* was significantly higher in the AC group than in the control group (4.3% vs. 3.4%; *P* = 0.038). The genus *Faecalibacterium* was not detected at 24 or 48 h but sharply increased at wk 1 to 12.7% in the AC group compared with only 2.5% in the control group; the relative abundance of this genus was similar between the 2 groups until wk 7 but declined in the control group by wk 8 compared with the AC group. Based on these data and Figure 3, an interaction between treatment and sampling point for *Faecalibacterium* relative abundance would be expected; however, the lmer model failed to detect such interaction effects due to the limited sample size. Although overall treatment differences were noted for *Faecalibacterium* and other genera, such findings warrant further investigation using a larger number of animals. Additionally, unclassified *Clostridiaceae* were more abundant in the AC group (*P* = 0.011) than in the control group, with a clear difference between the 2 treatment groups noted only at 24 h, whereas other sampling time points had similar relative abundance for the 2 treatment groups. However, changes in bacterial populations in response to treatments are associative in nature and therefore require further investigation to verify their clinical relevance and effect on animal performance.

**Figure 3:**
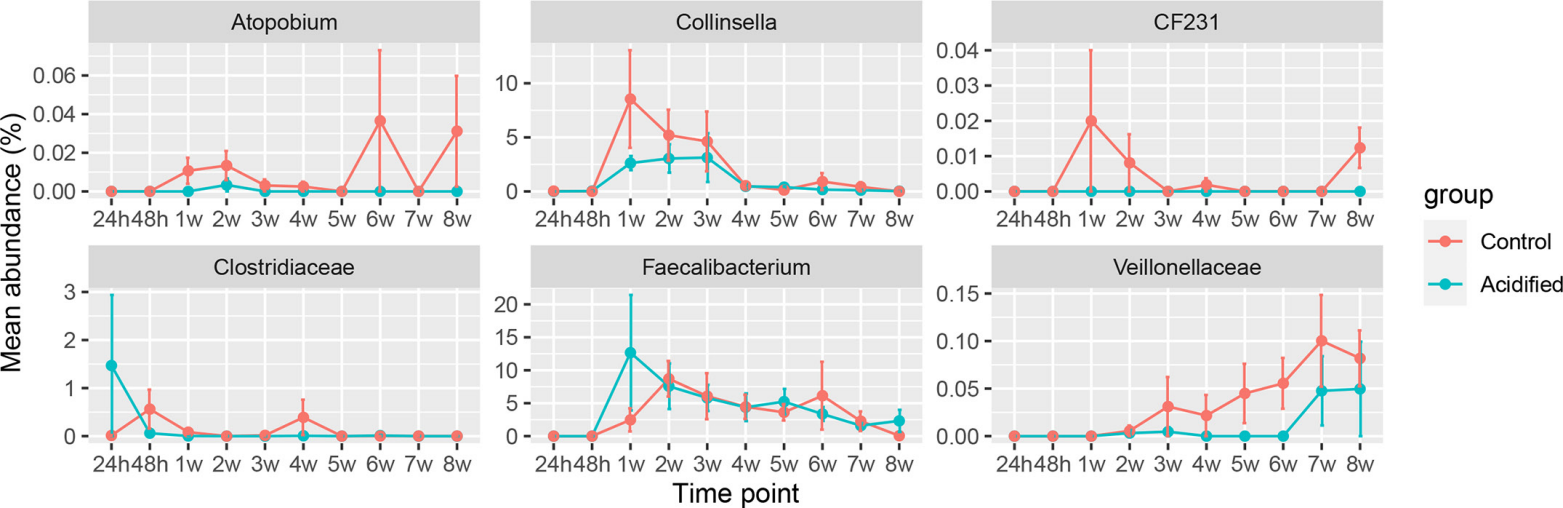
Relative abundance of 6 bacterial lineages that differed between the acidified colostrum and control groups across all sampling times. Statistical difference was evaluated by linear mixed effects model (lmer). Error bars represent the SEM. w = week.

In this study, we report changes in both community composition (α and β diversity) at 24 and 48 h in a cohort of dairy calves fed with similar diet and management. Such comparisons during the first week of life have been limited to a few reports (Alipour et al., 2018; Klein-Jöbstl et al., 2019; Virgínio Júnior et al., 2021). We report that bacterial richness was <50 ASV and Shannon diversity was <4 at 24 and 48 h, indicating that bacterial diversity at this age is limited to a handful of bacterial species that may have been acquired from the dam or the environment. Across both treatment groups, we observed a group of common bacterial lineages that contributed to most of the bacterial abundance; however, there was large variation in the commonly present abundant bacterial populations between the 2 groups. Further, we observed that treatment differences, although significant, were small compared with the differences observed between time points, indicating that acidification of colostrum did not induce wholesale shifts in microbial communities but influenced only a handful of bacterial populations.

Feeding acidified colostrum to calves induced shifts in a few bacterial populations at wk 1, with a spike in the relative abundance of *Faecalibacterium* in the AC group compared with the control group. In contrast, *Collinsella*, *Atopobium*, and *CF231* were more abundant in the control group than in the AC group. *Faecalibacterium* had a relative abundance of 12% at wk 1 and thereafter ranged from 4.4 to 7.0% before 5 wk. In Hennessy et al. (2020), we reported that the relative abundance of *Faecalibacterium* increased to 16.5% at wk 4 but then decreased as the calves aged. Therefore, feeding acidified colostrum followed by acidified milk in this study may have led to a higher relative abundance of *Faecalibacterium* as early as wk 1 that was sustained until wk 5, whereas feeding nonacidified colostrum followed by acidified milk did not lead to an increase in the relative abundance of *Faecalibacterium* populations until wk 2 in control calves. The higher relative abundance of *Faecalibacterium* was also associated with a numerically higher relative abundance of *Blautia* and *Dorea* from wk 1 through 3 in the AC group compared with the control group. However, both groups of calves had similar microbial profiles from wk 5 through the end of the study.

The putative positive benefits of higher relative abundance of *Faecalibacterium*, such as its association with improved weight gain (Oikonomou et al., 2013) and decreased incidence of diarrhea (Foditsch et al., 2015; Hennessy et al., 2021), have been reported in several studies. Feeding acidified waste milk to calves has also been shown to lead to a higher relative abundance of *Faecalibacterium* in both the mucosa- and digesta-associated bacteria in the cecum at 3 wk of age (Deng et al., 2017). The positive association between *Faecalibacterium* and *Blautia* was discussed in our previous report (Hennessy et al., 2020) and was also observed in this study. Foditsch et al. (2014, 2015) reported that growth of *Faecalibacterium* isolates is best achieved at pH 4.0 to 4.5 in vitro. Therefore, the feeding of acidified milk products to calves may support the growth of *Faecalibacterium* through this decrease in abomasal pH.

The bacterial strain *Faecalibacterium prausnitzii*, which makes up approximately 4% of human gut microbiota (Hold et al., 2003), has been proposed for use as a probiotic in humans (Martín et al., 2017) and in early stages of life in dairy calves (Foditsch et al., 2015). In calves, the prevalence of *Faecalibacterium* spp. during the first week of life has been strongly associated with improved live weight gain during the preweaning period (Oikonomou et al., 2013). In previous studies, *Faecalibacterium* was observed to be low at wk 1, to increase beginning at wk 2 with the greatest increase between wk 3 and 4, and then to gradually decrease by wk 7 before disappearing from the fecal microbiota (Foditsch et al., 2015; Hennessy et al., 2020). Diet is a driving factor; a higher allowance of whole milk was found to increase the relative abundance of *Faecalibacterium* and was positively associated with cecal butyrate and overall energy harvest in calves (Kumar et al., 2021). *Faecalibacterium prausnitzii* is a butyrate-producing bacterium (Duncan et al., 2002a) and produces several bioactive molecules such as shikimic and salicylic acids (Miquel et al., 2014, 2015) as well as microbial anti-inflammatory molecules that improve gut barrier function (Quévrain et al., 2016). Furthermore, Lenoir et al. (2020) discovered that *F. prausnitzii* has important roles in reducing different cancers via regulation of the Wnt/β-catenin signaling pathway and inhibits IL-8, a key proinflammatory cytokine. Administration of *F. prausnitzii* increased levels of the anti-inflammatory cytokine IL-10 in plasma and reduced stress-induced responses such as corticosterone, C-reactive protein, and IL-6 in rats (Hao et al., 2019). *Faecalibacterium* produces butyrate via the butyryl-CoA:acetate CoA-transferase pathway by consuming acetate (Duncan et al., 2002b), and our data demonstrate that acetate-producing bacteria such as *Blautia* and *Dorea* co-occur with *Faecalibacterium*.

This study has several limitations. The small sample size (10 total calves, 5/treatment group) makes interpretation of the clinical relevance of results difficult; however, with frequent sampling times (total of 10 sampling points), we were able to compare changes in bacterial populations over the entire experimental period. In addition, no markers of health such as growth parameters or fecal scores were analyzed in this study. Future research should include larger numbers of animals as well as analysis of health and growth parameters to better establish the link between acidification of colostrum and clinical outcomes, particularly those that explain the benefits associated with bacteria such as *Faecalibacterium*. Additionally, bacteria levels in acidified versus nonacidified colostrum were not measured and therefore it is not possible to determine whether the results were specifically the result of acidification or the result of decreased bacterial loads in the acidified colostrum. However, because *Faecalibacterium* requires a pH in the range of the acidified colostrum and only 5 additional bacterial genera showed differences between the 2 treatment groups, we posit that acidification of the colostrum, rather than differences in overall bacterial loads, was responsible for the results seen in this study. Also, acidification of only colostrum increased *Faecalibacterium* in AC compared with control at wk 1, but a switch to feeding acidified milk to all calves after colostrum feeding led to an increased relative abundance of *Faecalibacterium* from wk 2 across both groups. Future investigations involving randomized control studies with larger animal numbers are warranted to clarify the effect of acidification of colostrum on the gut microbiome and the associations between acidification of colostrum and health outcomes, and to compare the effects of other forms of colostrum preservation (such as pasteurization) with the results seen in this study.

As discussed above, previous reports (Oikonomou et al., 2013; Foditsch et al., 2015; Hennessy et al., 2021) indicate that *Faecalibacterium* is associated with improved growth and reduced incidence of diarrhea in the first few weeks of life in dairy calves. As the calf's diet and management are key to maintaining BW and production efficiency later in life, and as the gut microbiome plays an essential role during the neonatal period, it is critical to identify the microorganisms that play a role in maintaining good health during the first phase of life in dairy calves. Despite its stated limitations, this study reports that feeding acidified colostrum to dairy calves at birth may lead to early colonization of *Faecalibacterium*, possibly leading to significant improvements in growth and sustained health during the neonatal period.
